# AFLP analysis reveals a lack of phylogenetic structure within *Solanum *section *Petota*

**DOI:** 10.1186/1471-2148-8-145

**Published:** 2008-05-14

**Authors:** Mirjam MJ Jacobs, Ronald G van den Berg, Vivianne GAA Vleeshouwers, Marcel Visser, Rolf Mank, Mariëlle Sengers, Roel Hoekstra, Ben Vosman

**Affiliations:** 1 Biosystematics Group, Wageningen University and Research Centre, Wageningen, The Netherlands; 2 Plant Research International, Wageningen University and Research Centre, Droevendaalsesteeg 1, 6708PB Wageningen, The Netherlands; 3 Centre for Genetic Resources, Wageningen University and Research Centre, Wageningen, The Netherlands; 4 KEYGENE N.V., Wageningen, The Netherlands; 5 Plant Breeding, Wageningen University and Research Centre, Wageningen, The Netherlands; 6 Centre for BioSystems Genomics, P.O. Box 98, 6700 AB Wageningen, The Netherlands

## Abstract

**Background:**

The secondary genepool of our modern cultivated potato (*Solanum tuberosum *L.) consists of a large number of tuber-bearing wild *Solanum *species under *Solanum *section *Petota*. One of the major taxonomic problems in section *Petota *is that the series classification (as put forward by Hawkes) is problematic and the boundaries of some series are unclear. In addition, the classification has received only partial cladistic support in all molecular studies carried out to date.

The aim of the present study is to describe the structure present in section *Petota*. When possible, at least 5 accessions from each available species and 5 individual plants per accession (totally approx. 5000 plants) were genotyped using over 200 AFLP markers. This resulted in the largest dataset ever constructed for *Solanum *section *Petota*. The data obtained are used to evaluate the 21 series hypothesis put forward by Hawkes and the 4 clade hypothesis of Spooner and co-workers.

**Results:**

We constructed a NJ tree for 4929 genotypes. For the other analyses, due to practical reasons, a condensed dataset was created consisting of one representative genotype from each available accession. We show a NJ jackknife and a MP jackknife tree. A large part of both trees consists of a polytomy. Some structure is still visible in both trees, supported by jackknife values above 69. We use these branches with >69 jackknife support in the NJ jackknife tree as a basis for informal species groups. The informal species groups recognized are: Mexican diploids, Acaulia, Iopetala, Longipedicellata, polyploid Conicibaccata, diploid Conicibaccata, Circaeifolia, diploid Piurana and tetraploid Piurana.

**Conclusion:**

Most of the series that Hawkes and his predecessors designated can not be accepted as natural groups, based on our study. Neither do we find proof for the 4 clades proposed by Spooner and co-workers. A few species groups have high support and their inner structure displays also supported subdivisions, while a large part of the species cannot be structured at all. We believe that the lack of structure is not due to any methodological problem but represents the real biological situation within section *Petota*.

## Background

The secondary genepool of our modern cultivated potato (*Solanum tuberosum *L.) consists of a large number of tuber-bearing wild *Solanum *species which grow in various habitats from the southern states of the USA to the most southern parts of Chile and Argentina. These wild species are important as a resource for valuable traits that can be used to improve the quality of the cultivars, including resistance against important diseases like *Phytophthora infestans *and potato cyst nematodes (*Globodera *spp.). Therefore it is no surprise that the wild relatives of the cultivated potato have since long drawn the attention of many plant breeders and botanists. To benefit most from the possibilities that the secondary genepool has to offer, it is necessary to have a good insight in the taxonomy. The classical treatments of potato taxonomy are from Correll [1], and Hawkes [2], later followed by reviews from Spooner and Hijmans [3], Spooner and Salas [4], and van den Berg and Jacobs [5].

There are two major taxonomic problems in the section *Petota*. First, many described species are extremely similar to each other and section *Petota *seems to be overclassified [5]. In many cases, potato species can only be distinguished by means of multivariate analysis of quantitative characters and/or on the basis of geographic origin [6-9].

The main cause for these difficulties is the ability of many species in section *Petota *to hybridize easily with other species [4]. Many species have been suspected to arise from hybrid speciation. Other causes are high morphological similarity among species, and phenotypic plasticity in different environments [3]. In recent reviews the number of species is reduced due to increased insights in potato taxonomy. Hawkes [2] recognized 227 tuber bearing species (7 cultivated species included) and 9 non-tuber-bearing species within section *Petota*. Spooner and Hijmans [3] recognized 203 tuber-bearing species including 7 cultivated species. Finally, Spooner and Salas [4], reduced the number further to 189 species (including 1 cultivated species) in section *Petota*.

The second taxonomic problem is the series classification. Hawkes [2] classified section *Petota *into 19 tuber bearing series plus two non-tuber bearing series that vary considerably in the number of species included. The boundaries between some series are unclear. As outlined earlier by Spooner et al. [10], the series classification of Hawkes and previous authors has received only partial cladistic support in any molecular study to date. The cpDNA RFLP data from Spooner and Sytsma [11], Castillo and Spooner [12], Rodriguez and Spooner [13], and Spooner and Castillo [14] could only find support for a classification in 4 clades.

The aim of the present study is to focus on the second problem and to describe the structure within section *Petota*. In the present study the largest number of species and accessions to date are examined in one simultaneous AFLP analysis. The obtained data are used for evaluation of the hypothesis put forward by Hawkes [2] that section *Petota *can be divided in 21 series and the hypothesis of Spooner and Castillo [14], that the section consists of 4 clades only.

AFLP has proven to be a useful method to solve phylogenetic relationships at a low taxonomic level [15-17]. The application of AFLP has many advantages. It produces highly reproducible data [18], it does not need a priori sequence information and it has the ability of high resolution [17]. Because AFLP generates fragments at random over the whole genome it avoids the problem that many sequence data based phylogeny reconstructions have, e.g. the generation of a gene tree instead of a species tree [15].

## Results

### The large dataset (4929 genotypes)

**Figure 1:**
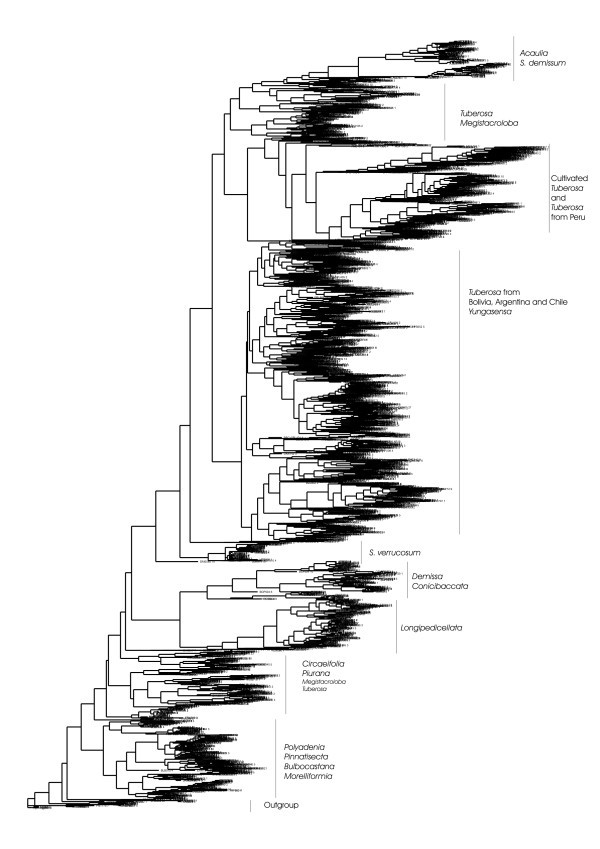
Neighbour Joining tree, complete dataset.

Figure 1 shows the Neighbor Joining (NJ) tree of the 4929 genotypes dataset. To describe the structure found in this NJ tree, we differentiate between 3 levels of structure: the accession level, the species level and the interspecies level.

At the accession level, the genotypes of the majority of the accessions cluster together. Of those accessions that do not form complete clusters, in most cases only one genotype deviates from the other 4 genotypes. In other cases, the accession was apparently so closely related with one or more other accessions that their genotypes formed a mixed group.

At the species level, 58 species or subspecies show consistency in their clustering, e.g. all accessions of a species cluster together. Nevertheless there are also many species (38 in total) whose accessions did not cluster all together and 48 species whose accessions were mixed with accessions of other species. The latter was often the case with species that occur in South America, the borders of many of these species are not clearly recognizable from the NJ tree.

Above the species level, a few clusters of species groups can be distinguished in the large NJ tree (but there is no indication on the statistical strength of the structure observed). Roughly, the following groups can be found in the NJ tree of the large dataset: 1) an outgroup with *S. nigrum, S. chaparense, S. sitiens, and S. fraxinifolium *2) North and Central American diploid series *Polyadenia*, *Pinnatisecta*, *Bulbocastana *and *Morelliformia*, 3) *Circaeifolia *and *Piurana *accessions, 4) *Longipedicellata *accessions, 5) *Demissa *and *Conicibaccata *accessions but without *S. demissum *and *S. semidemissum*, 6) *S. verrucosum *accessions, 7) *Tuberosa *from Bolivia, Argentina and Chile plus some accessions from other series such as *Yungasensa*, 8) accessions from cultivated *Tuberosa *species and wild *Tuberosa *from Peru, 9) accessions from *Tuberosa *and M*egistacroloba*, 10) accessions from *S. acaule *(and its subspecies), *S. albicans, S. demissum*, *S x semidemissum *and *S. edinense*.

### The condensed dataset (916 genotypes)

Because of the size of the dataset, it proved impossible to analyze it with cladistic methods nor to analyze it for statistical support. A condensed dataset was created by choosing a representative genotype from all the available accessions (see methods section for exact details). This condensed dataset consisted of 916 genotypes.

A single ratchet parsimony search consisting of 200 iterations yielded a Maximum Parsimony (MP) tree of 9669 steps. Furthermore, 20 individual independent ratchet searches each consisting of 50 iterations also yielded a MP tree of 9669 steps.

**Figure 2:**
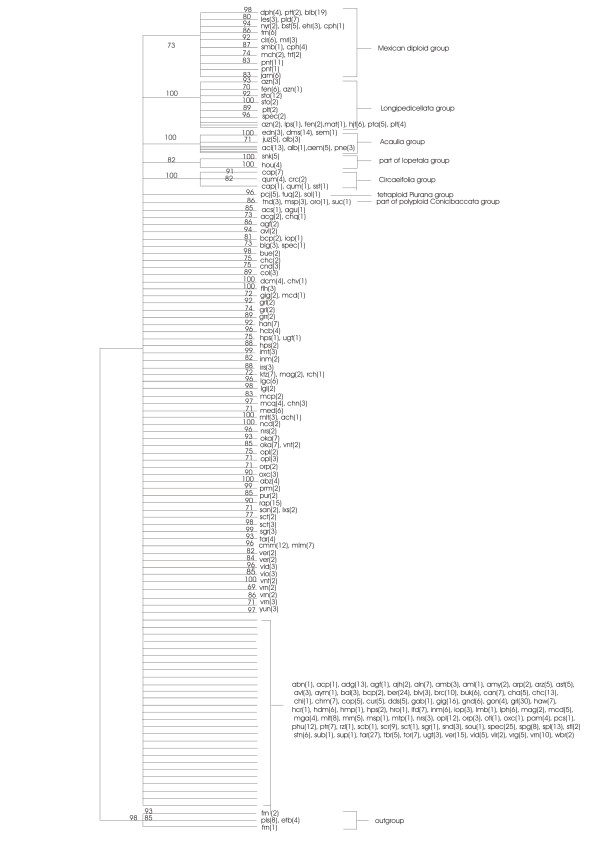
**Maximum Parsimony majority rule consensus tree, condensed dataset**. The numbers in parentheses indicate number of accessions. The numbers above the branches are Jackknife support values.

**Figure 3:**
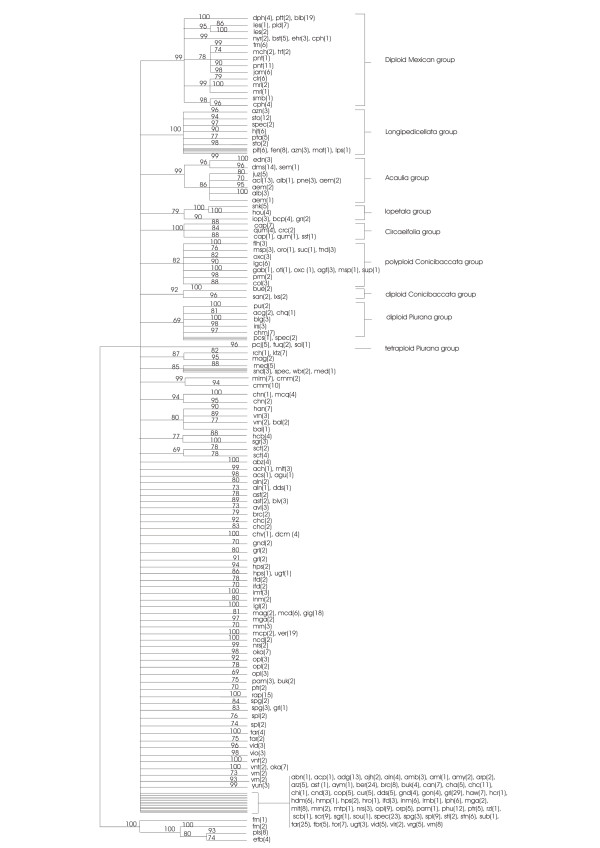
**Neighbour Joining majority rule consensus tree, condensed dataset**. The numbers in parentheses indicate number of accessions. The numbers above the branches are Jackknife support values.

Figure 2 shows the schematised majority rule consensus NJ jackknife tree and Figure 3 shows the schematised majority rule consensus MP jackknife tree of the condensed dataset. The strict consensus trees were manipulated in such a manner that not all the separate branches were represented but some were summarised. The schematised trees only show branches with more than 69 jackknife support. The original majority rule consensus NJ jackknife tree and majority rule consensus MP jackknife tree are available from the authors as supplemental data.

When comparing the NJ and the MP jackknife trees it is apparent that a large part of both trees consists of a polytomy. However, some structure is still visible in both trees, supported by jackknife values above 69. The following groups can be recognized in both the NJ jackknife tree and the MP jackknife tree:

1) Mexican diploid species, with a jackknife support of 73 for the MP tree and 99 for the NJ tree; the substructure found within the Mexican and Northern American diploids is almost the same for both trees.

2) A group of tetraploid Mexican/North and Central American species belonging to series *Longipedicellata*, with a jackknife support of 100 in both trees.

3) A group consisting of accessions of *S. acaule*, *S. demissum*, and closely related species with a jackknife support of 100 in the MP tree and 99 in the NJ tree.

4) A group consisting of the species belonging to series *Circaeifolia*, with a jackknife support of 100 in both trees.

5) A small group of accessions belonging to *S. paucijugum*, *S. tuquerrense*, and *S. solisii*, tetraploid species belonging to the series *Piurana*, with a jackknife support of 96 in the NJ tree and 92 in the MP tree.

There are also differences in group structure between the two trees. There are a number of groups that have good jackknife support in the NJ tree but are not supported in the MP jackknife tree:

1) A group of hexaploid Mexican species belonging to series *Demissa *with a jackknife support of 79. In the MP tree only 2 species that are part of this group were found in one small clade: *S. schenckii *and *S. hougasii*.

2) A group of accessions from species belonging to series *Conicibaccata *has a jackknife support of 82 in the NJ jackknife tree. In the MP jackknife tree the same accessions are part of the polytomy. These clades represent the subgroups found within the *Conicibaccata *group in the NJ tree. Only one subgroup is not represented by a similar clade in the MP jackknife tree.

3) A group of species belonging to series *Piurana *has a jackknife support of 69 in the NJ tree. In the MP tree, the jackknife support was low, so this group collapsed and 4 out of 5 supported subgroups found in the NJ jackknife tree are visible as supported separate small groups in the MP jackknife tree.

4) A group consisting of accessions from diploid species of series *Conicibaccata*, *S. buesii*, *S. sandemannii *and *S. laxissimum *with jackknife support of 92.

5) A group which contains accessions of *S. medians*, *S. sandemanii*, *S. weberbauerii *and an unknown species with a jackknife support of 85.

## Discussion

### The value of AFLP

One of the arguments against the use of AFLP is the possible bias caused by homoplasy [17,19,20]. Non-identical co-migrating bands in the AFLP fingerprints can contribute noise instead of signal to the dataset without being detected. However, it is not likely that in the tuber-bearing wild potatoes homoplasy will cause many problems because the species are all very closely related and homoplasy becomes a problem when distantly related species are involved. Koopman [16] showed that in a set of closely related *Lactuca *species, sufficient phylogenetic signal was present and concluded that in practice the influence of possible limitations of AFLP, such as co-migration of nonhomologous fragments is limited. However, he stresses that the conclusion only applies to datasets with closely related species. Moreover, Kardolus [19] concludes from his AFLP results that in *Solanum *section *Petota *the AFLP technique is suitable up to the species level. The AFLP method has since then successfully been used in more studies on potato taxonomy [21-24].

### Status of groups within section *Petota*

Not all the groups found in this study have the same level of cohesion or have the same level of demarcation. Some groups have clear borders, while from others we can only vaguely recognize the contours. First, there is a number of groups that are always well supported, whether the analysis is done in a phenetic or phylogenetic way, see Figure 2 and 3. This is the case for the group of Mexican diploid species, the group of Mexican tetraploids, the group of *S. demissum *and *S. acaule*, the group of *S. circaeifolium*, the group of *S. commersonii *and the group of *S. schenckii *and *S. hougasii*. Then there are groups that are not supported in the MP jackknife tree (Figure 2) but that can be found in both the original MP trees and NJ trees (not shown) and are supported in the NJ jackknife tree (Figure 3). This applies to the group with Mexican hexaploid species, the group containing polyploid species belonging to series *Conicibaccata*, the group containing diploid *Piurana *species, and the small groups of *S. huancabambense*, *S. kurtzianum*, *S. medians*, *S. mochiquense*, *S. hannemanii*, *S. buesii*, and *S. paucijugum*.

The largest part of the jackknife trees consists of a polytomy of species that does not seem to contain structure at all. If one was only to consider the structure shown in the jackknife trees, the conclusion would have to be that according to the results of the present AFLP analyses the largest part of section *Petota *is without any taxonomic structure.

However, it is possible to identify additional groups that are present in many of the original NJ and MP trees, but do not have enough support to be shown in the jackknife trees. For example, in the 4929 dataset NJ tree a cluster represents the group of cultivated potatoes together with species of series *Tuberosa *from Peru. The groups that are found in both the phenetic and phylogenetic analysis are strong groups with clear borders. The exchange of genetic material is most likely restricted to the members of the group. The groups with only low support in the MP alone or in both trees are groups that probably share a considerable amount of genetic material with genotypes outside the group. In a study of Jacobs, van den Berg and Vosman: Comparison of Chloroplast DNA and AFLP data from *Solanum *section *Petota *reveals incongruencies between the datasets, submitted, the incongruencies found between the chloroplast data and the AFLP data suggest that hybridization occurs between species of different series in section *Petota*. For example, the composition of species of the clade representing the series *Piurana *in the chloroplast tree is different from that of the clade representing the *Piurana *series in the AFLP tree.

The resulting groups also have implications for the theory on EBN of Hawkes and Jackson [25]. EBN stands for Endosperm Balance Number and refers to a hypothetical genetic factor that would explain the success or failure of crosses due to the functioning or breakdown of the endosperm after fertilization. Crosses between species with the same EBN are generally successful and crosses between species with different EBN generally are not, independent of ploidy levels. Hawkes and Jackson [25] claim that there is a correlation between the EBN hypothesis and the evolution of the group of tuber-bearing *Solanum *species. EBN 1 is found mainly in species that are considered to be close to the ancestors of the group: Mexican series *Morelliformia*, *Bulbocastana*, *Pinnatisecta*, and *Polyadenia*. The EBN 2 condition would have arisen as an isolating mechanism when potato species moved southwards. The EBN 4 condition occurs in hexaploids which are allopolyploids.

From the present results it is clear that there is no absolute relationship between EBNs and the groups found. In the group which contains *S. acaule*, *S. demissum*, *S. semidemissum *and *S. edinense*, different ploidy levels and different EBNs occur. This mixture of ploidy and EBN levels also occurs in the group with representatives of series *Conicibaccata*. The species *S. moscopanum *and *S. tundalomense *both are hexaploid and have EBN 4 and they form a group or cluster together with other series *Conicibaccata *species which are known to be tetraploid and have EBN 2. Although these tetraploid and hexaploid species from series *Conicibaccata *are mixed, the diploid series *Conicibaccata *(EBN 2) species do form a separate cluster.

With regard to the overall structure of the section as found in this study two main observations can be made. There seems to be a lack of supported structure, especially in the South American part of section *Petota*. Furthermore, there is a lack of support for the relationships between the different groups that were found in the NJ and MP trees. It is important to differentiate between these two phenomena because the causes underlying both cases could be different.

### Lack of structure in South American part of section *Petota*

The AFLP jackknife NJ tree and the jackknife MP tree in this study shows a lack of structure or rather, an unresolved structure for the part of the tree which contains South American species while the other part of the tree shows several well supported groups.

Kardolus et al. [19] mentioned that within series *Tuberosa *different genotypes of the same species are not always grouped together and are scattered among genotypes from other species. He claims that the cause of this phenomenon is not the lack of resolution of AFLP, but the overclassification of a group of species, the so-called brevicaule-complex. The cpDNA RFLP studies of Spooner and Sytsma [11], and Spooner and Castillo [14] also showed a lack of support for a resolved structure within the group of South American species, and the branch uniting all these species had a bootstrap support value of only 67.

Volkov et al. [26] compared the ETS region of rDNA for 30 species of *Solanum *section *Petota *and found high bootstrap values for the branch uniting all the South American species in three different types of dendrogram (Maximum parsimony, Bayesian statistics and Neigbour Joining). However, the two subgroups within the South American clade that they distinghuished (variants C1 and C2) often show polytomies and resolution within the groups is mostly lacking.

Outside the field of potato taxonomy, researchers have reported similar patterns. Hughes and Eastwood [27] report a low sequence divergence and lack of resolution in the large Andean clade of the genus *Lupinus*. This would point at a rapid and recent diversification in the Andes. The authors also suggest that *Lupinus *is probably only one example of many plant radiations that followed the final uplift of the Andes. They assume that many of these plant radiations are yet unknown. It is possible that the factors underlying the *Lupinus *diversification are also responsible for the *Solanum *section *Petota *diversification. According to Hughes and Eastwood [27] these factors would be the large scale of the area over which the radiation extends, repeated fragmentation of high altitude habitats due to quaternary climate fluctuations, the extremely dissected topography, and the habitat heterogeneity.

### Lack of support for relationships between different groups

Except for the outgroup consisting of *S. etuberosum*, *S. palustre *and *S. fernandezianum *which connects to the main branch of the NJ jackknife and MP jackknife tree with respectively 100 or 98 support value, none of the branches connecting two or more groups have jackknife support of 69 or higher. That is the reason why in the schematized jackknife NJ and jackknife MP trees these branches collapse in a polytomy. Contrastingly, the branches of the groups that can be recognised within the polytomy do have jackknife support, although not all species can be put in groups as discussed previously.

In the first study on the use of AFLP in *Petota *taxonomy by Kardolus et al. [19], it proved also difficult to find bootstrap support for branches connecting the different groups in section *Petota*. Bootstrap support above 70 were given for a NJ tree branch connecting the outgroup of *S. etuberosum *and *S. brevidens*, for a branch connecting the outgroups, and for the Mexican diploids and *S. circaeifolium *and *S. circaeifolium *subspecies *quimense *with the other part of the tree. In the cpDNA RFLP studies on the South American part of section *Petota *[14] only a few branches connecting the larger groups showed bootstrap support above 70. Clade 1, consisting of Mexican diploids (except *S. cardiophyllum *and *S. bulbocastanum*) is connected to the other clades with a bootstrap value of 87, and Clade 3 (mainly accessions belonging to series *Piurana*) and Clade 4 (the rest of section *Petota*) are connected to each other with a branch with 96 bootstrap support.

We can conclude from these previous results that it is indeed difficult to find good support for the backbone structure of section *Petota *in general. This indicates that our and previous results represent the real biological situation in *Solanum *section *Petota*. Since the phylogenetic signal is clearly present in our data as shown in the well-supported groups in the present study, the lack of structure in parts of the tree is not caused by the lack of phylogenetic signal in AFLP markers.

### New informal species groups for *Solanum *section *Petota*

As outlined in this paper and in other earlier studies, there are no results that support the classification of section *Petota *in 21 series. Although a few of the series seem to form natural groups, the majority of the series as proposed by Hawkes [2] could not be found as separate clusters or clades. Our goal is to use the found structure in the present study at maximum for classifying the section *Petota*.

We propose to divide section *Petota *in informal species groups, following the approach of Spooner et al. [10] who constructed 11 informal species groups for the North and Central American species. They followed the approach of Whalen [28] and Knapp [29,30] who applied a similar informal species group classification. We will use the names already used by Spooner et al. [10] if applicable, and add new groups that were not treated in their study. We chose to base the informal group classification on the groups that are supported in the NJ jackknife tree. The NJ jackknife tree shows more resolution relative to the MP. However, it would not be useful to consider every small group that appears in the schematized tree as a biologically meaningful group. Therefore, the choice for species groups is restricted to groups of species that make sense in the light of former studies and contain at least 3 species. We maintain the species group Verrucosa which contains only one species, because this species group is already designated by Spooner et al [10].

In total, the NJ jackknife tree can be partitioned into 10 species groups. It would be possible to construct more species groups based on the structure shown in the various trees made in the present study, but these groups would then not be supported by bootstrap or jackknife supports.

Although a closed classification following the rules of the Botanical Code is desirable, it seems in this case difficult to apply. In the present study, many species cannot be accommodated in groups. These species do not automatically form a group themselves, but are intentionally left unclassified.

We suggest recognizing the following informal species groups as shown in the NJ jackknife tree (Figure 3):

#### Diploid Mexican group

This group contains the species groups of Spooner et al. [10]: Pinnatisecta, Stenophyllidia, Trifida, Polyadenia, Morelliforme, and Bulbocastana. These species groups can be recognized in the present study as separate branches within the NJ cluster which represents this species group. In the present study we recognize a higher level of group structure which contains all the mentioned species groups, because the detailed contents of each subgroup in our study (Figure 3) differs from the contents from the species groups from Spooner et al. [10].

#### Acaulia group

In our study this group contains 2 supported subgroups, one branch with jackknife support of 96 containing the species *S. semidemissum*, *S. demissum *and *S. x edinense*. The other group shows a jackknife support of 98 and contains *S. juzepczukii*, *S*. *albicans *and the three subspecies *S. acaule *subsp. *acaule*, *S. acaule *subsp. *aemulans*, *S. acaule *subsp. *punae*.

#### Iopetala group

This group contains the species *S. schenckii*, *S. hougasii*, that form a strongly supported cluster together (jackknife support 100) and a cluster containing the species *S. iopetalum*, *S. brachycarpum*, *S. guerreroense *(jackknife support 90). All species were formerly designated by Hawkes [2] to series *Demissa *which also included the species *S. demissum *and closely related species. The species in our group are the same as in the species group Iopetala designated by Spooner et al. [10]. They reduced the species *S. brachycarpum *as a synonym of *S. iopetalum*.

#### Longipedicellata group

As the name does suggest, this group contains species that were formerly placed by Hawkes [2] in the series of *Longipedicellata*. The species included in this group are *S. fendleri *including *S. fendleri *subsp. *arizonicum*, *S. stoloniferum*, *S. hjertingii*,. *S. papita*, *S. polytrichon*, *S. leptosepalum*, and *S. matehualae*. The species *S. leptosepalum*, *S. fendleri*, *S. papita*, and *S. polytrichon *have been reduced as synonyms of *S. stoloniferum *[10]. The species *S. matehualae *is reduced as synonym of *S. hjertingii *[10].

#### Polyploid Conicibaccata group

This group contains species placed there by Spooner et al. [10], complemented with South American species. The species in this species group are mainly the same as Hawkes [2] placed in series *Conicibaccata*. According to the present study the group consists of *S. flahaultii*, *S. moscopanum*, *S. orocense*, *S. sucubunense*, *S. tundalomense*, *S. oxycarpum*, *S. longiconicum*, *S. garcia-barrigae*, *S. otites*, *S. oxycarpum*, *S. agrimonifolium*, *S. moscopanum*, *S. subspanduratum*, *S. paramoense*, and *S. colombianum*.

#### Diploid Conicibaccata group

Although most of the series *Conicibaccata *can be put in the species group Conicibaccata there are a few species that form a separate group. This group consists of the diploid species *S. buesii, S. sandemanii*, and *S. laxissimum*.

#### Diploid Piurana group

This species group was not designated by Spooner et al. [10]. The name refers to the former series *Piurana *as the contents of the group are roughly similar: *S. piurae*, *S. acroglossum*, *S. blanco-galdosii*, *S. irosinum*, *S. chomatophilum*, and *S. paucissectum *from series *Piurana *and *S. chiquidenum *from series *Tuberosa*.

#### Tetraploid Piurana group

The situation as described before for the Conicibaccata group also applies partly for the Piurana group. There are a few species from the formerly designated *Piurana *series [2] that form their own species group. This species group contains the tetraploid species *S. paucijugum*, *S. tuquerrense*, and *S. solisii*.

#### Circaeifolia group

This group consists of *S. circaeifolium*, *S. soestii*, *S. capsicumbaccatum *and *S*. *circaeifolium *subsp. *quimense*. The contents is conform Hawkes' series *Circaeifolia*.

#### Verrucosa group

This group contains only 2 species; *S. macropilosum *and *S. verrucosum*. The species *S. macropilosum *was reduced to a synonym of *S. verrucosum *by Spooner et al. [10].

## Conclusion

As far as we know, this paper treats the largest collection of *Solanum *section *Petota *accessions ever analysed simultaneously. All other previous studies used datasets that included less variation and fewer species. Because of the thorough sampling, it is possible to propose species groups without too many reservations. A number of species groups coincide with certain series recognized by Hawkes [2]. However, most of the series that Hawkes and his predecessors recognized, cannot be supported any longer as natural groups, based on our current knowledge. The present study shows that the taxonomic structure of *Solanum *section *Petota *is highly unbalanced. A few species groups have high support and their inner structure displays also supported subdivisions, while a large part of the species cannot be structured and they seem to be all equally related to each other and to the supported groups.

It might be difficult to accept that a part of genus *Solanum *section *Petota *cannot be structured or subdivided. We even doubt that it would be possible to find more resolution with other methods or more markers, and we consider it likely that the polytomy is indicative of the real situation in section *Petota*. A relatively fast spread of tuber-bearing *Solanum *species over South America, due to the geographic conditions in the Andes [27], combined with high levels of hybridisation may explain why the phylogenetic links between species are so difficult to establish.

## Methods

### Plant Material

In total 951 accessions representing 196 different taxa, species, 15 subspecies and 17 hybrids were sampled. We tried to include as many species as possible from various gene banks. In principle, at least 5 accessions from each available species and 5 individual plants per species (totally approx. 5000 genotypes) were included. Seeds were surface-sterilized and sown in vitro at 25°C. The collection of individual *Solanum *clones was grown in vitro for at least 6 weeks on MS medium supplemented with 20% sucrose [31] at 18°C. DNA was extracted from leafs according to the method described by Stewart and Via [32].

### Nomenclature

Additional file 1 lists the species used and the accessions representing the species names according to the passport information from the gene bank. The labels used are not corrected according to the synonymy in recent taxonomic revisions for two reasons. First, we do not want to change an original label of an accession without actually checking the identity of that accession. Furthermore, by retaining the original labels it is possible to check many hypotheses on the taxonomy of species. However, we have included some remarks about recent taxonomy changes in Additional file 1. In some cases names/labels were corrected by us after preliminary AFLP results and visual inspection of the plant material in the greenhouse or on the field. If an accession could be assigned to another species according to AFLP pattern and morphology, it was given the name of this species, if there were any doubts on the identification the species was given the label *S. spec.*. The accessions which labels were changed are indicated in Additional file 1.

### AFLP

The samples were fingerprinted with two *Eco*RI/*Mse*I AFLP primer combinations: E32/M49 and E35/M48. The protocol of Vos et al. [33] was used to generate AFLP fragments. Primer combination E32/M49 yielded 91 polymorphic bands and primer combination E35/M48 yielded 131 bands. Keygene carried out the AFLP analysis on a MegaBACE 2.1 and scored the bands using their proprietary software. Bands were scored as dominant markers, so only the presence or the absence of a band was scored.

### Datasets

The dataset in this study originally contained 4929 genotypes. This large dataset was analyzed with NJ and UPGMA. Because of the size of the dataset, it proved impossible to analyze it with cladistic methods nor to analyze it for statistical support, even using the SARA supercomputer (see below). It was sheer impossible for a personal computer to do any further analyses apart from the NJ and UPGMA, and for the SARA computer cluster it would have taken many months/years of computing time.

For further analysis a condensed dataset was created by carefully choosing a representative genotype from all the available accessions. This condensed dataset consisted of 916 genotypes. The condensed dataset was used in both phenetic and cladistic analyses and in the resampling methods.

Besides choosing only one genotype per accession to represent the accession in the condensed dataset, other adjustments were made to create this dataset. All the 22 known interspecific hybrid accessions were removed, 23 other accessions were completely removed because of the extreme heterogeneity of the accession (possibly resulting from a mixture of species) in both the NJ and the UPGMA trees. Species labels of 49 accessions were changed based on their position in the NJ and/or UPGMA tree (not shown) and visual inspection of the plants in the experimental field or greenhouse in 2005 and 2006. In total 11 outgroup accessions were removed because preliminary AFLP results showed these outgroups to be too distant (*S. sitiens, S. nigrum, S. chaparense, S. lycopersicoides, S. canense, S. fraxinifolium*). The outgroup species *S. etuberosum*, *S. palustre *and *S. fernandezianum *were retained in the dataset.

### Data analysis

Both the phenetic and the cladistic analyses were conducted using PAUP 4.0 Altivec [34] on the TERAS computing cluster of SARA computing facilities in Amsterdam. For the 4929 phenetic analysis we used the total character distance, for the 916 data set we used the NeiLi distance [35] to calculate the distance matrix. A Neighbor Joining Jackknife tree was calculated using 10.000 replicates.

The cladistic analysis heuristic searches were done by using PRAP, Parsimony Ratchet Analyses using PAUP, a program that writes commands for PAUP. The commands in PRAP describe how PAUP should carry out parsimony ratchet searches [36]. By using parsimony ratchet, as described by Nixon [37], many tree islands are searched instead of thoroughly searching through each island.

For the MP jackknife analysis, we followed the conclusions drawn by Muller [36] that using random addition sequence instead of simple addition sequence has no beneficial effect on bootstrap or jackknife support. Also, a jackknife or bootstrap analysis using one heuristic search saving one tree per jackknife replicate and simple addition sequence, performed as good as or even better than an analysis using 10 parsimony ratchet iterations using the shortest tree only or using a strict consensus tree of all shortest trees [36]. Therefore, we conducted a jackknife MP analysis by performing 10.000 replicates using simple addition, and saving one shortest tree per replicate.

## Authors' contributions

MJ carried out the phenetic and phylogenetic analyses and drafted the manuscript, RVB and BV participated in coordination and design of the study and participated in drafting the manuscript, RH chose the plant material and participated in coordination and design of the study, VV and MV coordinated and carried out the growing of the material, MS and RM carried out the AFLP analyses. All authors read and approved of the final manuscript.

## Supplementary Material

Additional file 1 List of material used in the AFLP analyses. List of material used in the AFLP analyses. **Symbols used in additional file 1**. **# **recorded hybrid, removed in 916 dataset. **$ **complete accession removed in the 916 dataset because of conflicting positions in NJ tree. **&** removed outgroups in 916 dataset: *S. lycopersicoides*, *S. nigrum*, *S. chaparense*, *S. sitiens*, *S. canense*, *S. fraxinifolium*. * the label of this accession was changed in the 916 dataset after checking the position in the large NJ tree and checking morphology in the greenhouse/field. () the number in parentheses indicates the number of accessions used for the 916 analysis in case of removal or change of accessions. Abbreviations for Genebank source codes: CPC: Commonwealth Potato Collection, UK. CGN: Centre for Genetic Resources, the Netherlands. cgn: cgn receipt number, Centre for Genetic Resources, the Netherlands. PI: Plant Introduction number, USA. GLKS: Gross Lusewitz, Germany. Click here for file
