# Supplementary material for: AFLP analysis reveals a lack of phylogenetic structure within Solanum section Petota
Source: BMC Evol Biol. 2008 May 14;8:145. doi: 10.1186/1471-2148-8-145 (PMC2413236; doi:10.1186/1471-2148-8-145)
Supplement: Additional file 1 — List of material used in the AFLP analyses. List of material used in the AFLP analyses. Symbols used in additional file 1. # recorded hybrid, removed in 916 dataset. $ complete accession removed in the 916 dataset because of conflicting positions in NJ tree. & removed outgroups in 916 dataset: S. lycopersicoides, S. nigrum, S. chaparense, S. sitiens, S. canense, S. fraxinifolium. * the label of this accession was changed in the 916 dataset after checking the position in the large NJ tree and checking morphology in the greenhouse/field. () the number in parentheses indicates the number of accessions used for the 916 analysis in case of removal or change of accessions. Abbreviations for Genebank source codes: CPC: Commonwealth Potato Collection, UK. CGN: Centre for Genetic Resources, the Netherlands. cgn: cgn receipt number, Centre for Genetic Resources, the Netherlands. PI: Plant Introduction number, USA. GLKS: Gross Lusewitz, Germany. [file 1471-2148-8-145-S1.pdf]

series according to Hawkes (1990)	species	species abbreviation	accessions code	source codes (genebank)	total nr. of accessions in 4929 dataset (total nr. of accessions in 916 dataset)
Tuberosa II	*S. abancayense* Ochoa	abn	423	CGN 18357	1 (1)
Acaulia	*S. acaule* Bitter	acl	256, 317, 318, 424, 425, 426, 427, 428, 429, 430, 431, 432, 513*, 681*, 861*	CPC 3768, CGN 17843, CGN 20620, CGN 18179, CGN 18203, cgn962297, CGN 22768, CGN 18068, CGN 22332, CGN 21328, CGN 17930, CGN 22359, CGN 21366*, CGN 20623*, CGN 17924*	13 (13)
Acaulia	*S. acaule subsp. aemulans* (Bitter and Wittm.) Hawkes and Hjert.	aem	434, 435, 436, 437, 669*	CGN 21303, CGN 21330, CGN 21331, CGN 23789, CGN 20562*	5 (4)
Acaulia	*S. acaule subsp. punae* (Juz.) Hawkes and Hjert.	pne	441, 442, 443	CGN 20672, CGN 20665, CGN 20669	3 (3)
Tuberosa III	*S. achacachense* Cárdenas	ach	99	GLKS 32830	1(1)
Piurana	*S. acroglossum* Juz.	acg	45, 448	PI 498204, PI 365313	2(2)
Tuberosa II	*S. acroscopicum* Ochoa	acs	100	GLKS 32436	1 (1)
Conicibaccata	*S. agrimonifolium* Rydb.	agf	101, 319, 450	GLKS 32292, CGN 18285, CGN 22356	3 (3)
Tuberosa III	*S. ajanhuiri* Juz. and Bukasov	ajh	47$, 453, 454	PI 611096$, CGN 18239, CGN 22389	3 (2)
Tuberosa III	*S. alandiae* Cárdenas	aln	257, 320, 455, 457, 458, 459, 460	CPC 7212, CGN 18245, CGN 22349, cgn962384, CGN 20651, CGN 18260, CGN 18264	7 (7)
Acaulia	*S. albicans* (Ochoa) Ochoa	alb	301, 461, 462, 464,	CIP 761605, CGN 20667, CGN 20670, CGN 20674	4 (4)
Piurana	*S. albornozii* Correll	abz	2, 102, 103, 466	PI 561637, GLKS 35297, GLKS 35298, CGN 22731	4 (4)
Tuberosa II	*S. amabile* Vargas	aml	3	PI 365356	1 (1)
Tuberosa II	*S. amayanum* Ochoa	amy	302, 303	CIP 763004, CIP 763005	2 (2)
Tuberosa II	*S. ambosinum* Ochoa	amb	104, 105, 467,	GLKS 32282, GLKS 35299, CGN 18358	3 (3)
Tuberosa II	*S. ancophilum* (Correll) Ochoa	acp	304	CIP 761448	1 (1)

Megistacroloba	*S. aracc-papa* Juz.	arp	109, 110	GLKS 30082, GLKS 30081	2 (2)
Yungasensa	*S. arnezii* Cárdenas	arz	4, 111, 112, 113, 471	PI 545880, GLKS 32832, GLKS 32833, GLKS 32834, GLKS 32831	5 (5)
Yungasensa / Tuberosa	*S. arnezii x S. hondelmanii*	arz x hdm	401#	CGN 18191#	1 (0)
Megistacroloba	*S. astleyi* Hawkes and Hjert.	ast	114, 472, 474, 475, 476	GLKS 32836, CGN 18207, CGN 18210, CGN 18211, CGN 18212	5 (5)
Tuberosa II	*S. augustii* Ochoa	agu	305	CIP 762631	1 (1)
Tuberosa III	*S. avilesii* Hawkes and Hjert.	avl	477, 478, 479,	CGN 18255, CGN 18256, CGN 18257	3 (3)
Tuberosa II	*S. aymaraesense* Ochoa	aym	5	PI 607896	1 (1)
Tuberosa III	*S. berthaultii* Hawkes	ber	322, 323, 324, 480, 481, 482, 483, 484, 485, 486, 487, 488, 489, 490, 491, 492, 493, 494, 561*, 939, 940, 941, 943, 944	CGN 20644, CGN 20650, CGN 18042, CGN 18074, CGN 18190, CGN 20635, CGN 20636, CGN 22715, CGN18216, CGN 22716, CGN 20645, CGN 18246, CGN 23804, CGN 18228, CGN 22727, BGRC 15479, CGN 17823, CGN 18118, GLKS 31670*, CGN 18189, CGN 23508, CGN 18267, CGN 17716, CGN 23477	23 (24)
Tuberosa III / Tuberosa II	*S. berthaultii x S. sparsipilum*	ber x spl	402#	CGN 18229#	1 (0)
Piurana	*S. x blanco-galdosii* Ochoa	blg	48, 115, 996	PI 442701, GLKS 35309, CIP 761051	3 (3)
Megistacroloba	*S. boliviense* Dunal	blv	496, 498, 499	CGN 18196, CGN 18070, INTA 73228B	3 (3)
Pinnatisecta	*S. brachistotrichium* Bitter (Rydb.)	bst	116$, 117, 118, 258, 325, 500,	GLKS 32717$, GLKS 32801, GLKS 32714, CPC 3822, CGN 17681, CGN 17603	6 (5)
Demissa	*S. brachycarpum* Correll	bcp	259, 321*, 326, 501, 504$	CPC 7028, CGN 18344*, CGN 17721, GLKS 31686, CGN 18347$	4 (4)
Tuberosa III	*S. brevicaule* Bitter	brc	327, 505, 506, 507, 509, 1020, 1025, 1026, 1040, 1047,	CGN 18231, CGN 17841, CGN 18226, CGN 18232, CGN 22321, CGN 18030, CGN 18223, CGN18247, CGN22322, CGN22717	10 (10)
Tuberosa III / Tuberosa II	*S. brevicaule x S. sparsipilum*	brc x spl	403#, 404#	cgn962403#, cgn962443#	2 (0)
Conicibaccata	*S. buesii* Vargas	bue	7, 8	PI 568922, PI 607889	2 (2)

Tuberosa II	*S. bukasovii* Juz.	buk	328, 511, 512, 514, 955, 971,	CGN 17683, CGN 17684, CGN 17737, CGN 17821, CGN 21305, CGN 17738	6 (6)
Bulbocastana	*S. bulbocastanum* Dunal	blb	330, 331, 515, 516, 517, 518, 519, 520, 521, 522, 523, 524, 525, 945, 946, 947, 948, 949, 950	CGN 21306, CGN 17693, CGN 17690, CGN 17691, CGN 22698, CGN 17692, cgn960631, CGN18310, CGN17687, GLKS 31741, CGN 21363, CGN 21364, CGN 23075, CGN 17689, CGN 22367, pi275199, CGN 23010, CGN 23074, CGN 22732	19 (19)
Bulbocastana	*S. bulbocastanum* subsp. *dolichophyllum* (Bitter) Hawkes	dph	9, 10, 119, 937	PI 255516, PI 545752, GLKS 35399, CGN 17688	4 (4)
Bulbocastana	*S. bulbocastanum* subsp. *partitum* (Correll) Hawkes	ptt	120, 121	GLKS 35322, GLKS 35323	2 (2)
Tuberosa II	*S. cajamarquense* Ochoa	cjm	122$	GLKS 35328$	1 (0)
Tuberosa II	*S. canasense* Hawkes	can	260, 526, 527, 528, 529, 951$, 952, 953	CPC 2725, cgn960639, CGN 17722, CGN 17672, CGN 17589, CGN 20592$, CGN 18072, CGN23007	8 (7)
Tuberosa III	*S. candolleanum* P. Berthault	cnd	530, 531, 532	PI 498226, CGN 18132, CGN 20603	3 (3)
Canensa, subsect. Basarthrum	*S. canense* Rydb.	cns	332&, 333&	CGN 18062&, CGN 18123&	2 (0)
Circaeifolia	*S. capsicibaccatum* Cárdenas	cap	261, 334, 335, 534, 535, 536, 537, 538,	CPC 3554, CGN 18297,CGN 18268, CGN 18291, CGN 18254, CGN 22388, cgn962457, CGN 18265	8 (8)
Cardiophylla	*S. cardiophyllum* Lindley	cph	124$, 336, 337, 539, 541, 542	GLKS 30099$, CGN 18325, CGN 18326, BGRC 55227, CGN 22387, CGN 17697	6 (5)
Yungasensa	*S. chacoense* Bitter	chc	125, 126, 127, 246*, 263, 338, 470$, 543, 544, 545, 546, 547, 548, 549, 550, 551	GLKS 30162, GLKS 30161, GLKS 30180, GLKS 32343*, CPC5901, CGN 18248, CGN 17679$, cgn962709, CGN 18365, CGN 17702, CGN 22384, CGN 18202, CGN 18294, CGN 18338, cgn961764, CGN 22368	15 (15)
Tuberosa II	*S. chancayense* Ochoa	chn	1, 552, 553	VIR 20892, CGN 18036, CGN 18356	3 (3)
	S. chaparense	chp	339&	CGN 18060&	1 (0)

Tuberosa III	*S. chaucha* Juz. and Bukasov	cha	128, 129, 130, 131, 132	GLKS 30125, GLKS 30115, GLKS 30120, GLKS 30118, GLKS 30119	5 (5)
Megistacroloba	*S. chavinense* Correll	chv	11	PI 498235	1 (1)
no information	*S. chillonanum* Ochoa	chi	12	PI 607890	1 (1)
Tuberosa II	*S. chiquidenum* Ochoa	chq	49$, 50, 51$	PI 310989$, PI 473459, PI 310942$	3 (1)
Conicibaccata	*S. chomatophilum* Bitter	chm	555, 556, 557, 558, 559, 562, 997	BGRC 7171, BGRC 18519, CGN 18037, CGN 17712, CGN 20574, CGN 17713, cgn960685	7 (7)
Circaeifolia	*S. circaeifolium* Bitter	crc	563, 564	BGRC 27058, CGN 18133	2 (2)
Circaeifolia	*S. circaeifolium subsp. quimense* Hawkes and Hjert.	qum	340, 341, 565, 566, 567,	CGN 18127, CGN 18128, CGN 20643, CGN 22767, CGN 18158	5 (5)
Bulbocastana	*S. clarum* Correll	clr	52, 133, 568, 569, 570, 999	PI 275202, GLKS 32378, cgn962759, cgn962765, cgn962773, cgn962782	6 (6)
Tuberosa II	*S. coelestipetalum* Vargas	cop	134, 135, 306, 307, 572	GLKS 35433, GLKS 35434, CIP 761755, CIP 761999, CGN 20557	5 (5)
Conicibaccata	*S. colombianum* Bitter	col	136, 137$, 573, 574	GLKS 31536, GLKS 31530$, CGN 18287, CGN 18289	4 (3)
Commersoniana	*S. commersonii* Dunal	cmm	265, 575, 576, 577, 578, 1017, 1018, 1019, 1027, 1028, 1039, 1050	CPC 5861, cgn961592, cgn961597, CGN 18027, CGN 22351, CGN 17988, CGN 18024, CGN 18026, CGN 18327, CGN 18328, GLKS 35340, CGN 23492	12 (12)
Commersoniana	*S. commersonii* subsp. *malmeanum* (Bitter)	mlm	139, 266, 579$, 580, 581, 1038, 1045, 1058	GLKS 35340, CPC 7520, CGN 18329$, CGN 18025, CGN 18215, CGN21353, CGN 22352, cgn962274	8 (7)
Tuberosa III	*S. curtilobum* Juz. and Bukasov	cur	140, 141, 142, 143, 267	GLKS 31620, GLKS 31628, GLKS 35346, GLKS 31627, CPC 7323	5 (5)
Demissa	*S. demissum* Lindley	dms	264*, 299*, 342, 343, 344, 345, 364*, 510*, 582, 584, 585, 586, 587, 748$, 1060	CPC 5858*, CPC 7069*, CGN 18313, CGN 17820, CGN 20571, CGN 17810, CGN 17829*, cgn960640*, CGN 17797, CGN 17794, CGN 18302, CGN 22378, CGN 20570, CGN 23062$, CGN 17800	15 (14)

Tuberosa III	*S. x doddsii* Correll	dds	144, 145, 146, 588, 589,	GLKS 32882, GLKS 32883, GLKS 32880, CGN 20661, CGN 18359	5 (5)
Tuberosa II	*S. dolichocremastrum* Bitter	dcm	147, 148, 149, 308,	GLKS 32342, GLKS 35348, GLKS 35349, CIP 762533	4 (4)
Demissa	*S. x edinense* P. Berthault	edn	150, 151, 152	GLKS 25493, GLKS 25492, GLKS 25494	3 (3)
Pinnatisecta	*S. cardiophyllum* subsp. *ehrenbergii* Bitter	ehr	153, 154, 155,	GLKS 35331, GLKS 35332, GLKS 32158	3 (3)
Etuberosa	*S. etuberosum* Lindl.	etb	591, 593, 594, 595	CGN 17714, CGN 23066, CGN 18242, CGN 20648	4 (4)
Longipedicellata	*S. fendleri* A. Gray	fen	156, 157, 158, 159, 160, 596, 597, 598	GLKS 30425, GLKS 30428, GLKS 30429, GLKS 30433, GLKS 30444, CGN 18116, CGN 18063, CGN 17715	8 (8)
Longipedicellata	*S. fendleri* subsp. *arizonicum* Hawkes	azn	13, 54, 55, 56, 161, 162	PI 497996, PI 497999, PI 498000, PI 498001, GLKS 35350, GLKS 35351	6 (6)
Etuberosa	*S. fernandezianum* Phil.	frn	59, 599, 600,	PI 566756, CGN 18360, CGN 18243	3 (3)
Conicibaccata	*S. flahaultii* Bitter	flh	14, 15, 61	PI 570620, PI 583306, PI 583317	3 (3)
Suaveolentia subgenus Basarthrum	*S. fraxinifolium*	frx	602&	CGN 21368&	1 (0)
Tuberosa III	*S. gandarillasii* Cárdenas	gnd	16, 62, 163, 270, 346, 603,	PI 597750, PI 597751, GLKS 32423, CPC 7044, CGN 20560, CGN 17590	6 (6)
Conicibaccata	*S. garcia-barrigae* Ochoa	gab	17	PI 498158	1 (1)

Tuberosa III	*S. gourlayi* Hawkes	grl	347, 604, 605, 606, 607$, 608, 609, 610, 611, 1000$, 1005, 1006, 1008, 1009, 1010, 1011, 1012, 1013, 1014, 1015, 1021, 1022, 1029, 1030, 1032, 1033, 1034, 1035, 1036$, 1037, 1042, 1043$, 1044, 1048, 1049, 1051, 1052$, 1053, 1054, 1055$	CGN 17851, CGN 22705, CGN 17591, CGN 18039, CGN 22380$, cgn961345, CGN 17592, CGN 22336, CGN 21335, cgn961607$, CGN 17872, CGN 17873, CGN 17962, CGN 17963, CGN 17965, CGN 17966, CGN 17967, CGN 17969, CGN 17970, CGN 17971, CGN 18065, CGN 18066, CGN 20585, CGN 20594, CGN 20657, CGN 21332, CGN 21333, CGN 21334, CGN 21336$, CGN 21341, CGN 22340, CGN 22342$, CGN 22343, CGN 23022, CGN 23486, CGN 23497, CGN 23515$, cgn960071, cgn961347, CGN 23794$	40 (34)
Tuberosa III	*S. gourlayi subsp. pachytrichum x S. leptophyes*	ptr	612, 613, 614, 615, 616, 617, 618,	cgn18102, cgn18176, bgrc27294, bgrc27295, cgn18188, bgrc7231, bgrc28084	7 (7)
Tuberosa III	*S. gourlayi* subsp. *pachytrichum x S. leptophyes*	ptr x lph	409#	BGRC 27293#	1 (0)
Tuberosa III	*S. gourlayi* subsp. *vidaurrei (Cárdenas) Hawkes and hjert.*	vid	619, 620, 621, 622, 623, 624, 625, 626,	CGN 17848, CGN 17849, CGN 18040, CGN 17850, CGN 18038, CGN 17864, CGN 23024, CGN 23045	8 (8)
Demissa	*S. guerreroense* Correll	grr	348, 627	CGN 18290, GLKS 31513	2 (2)
	S. hannemanii	han	252*, 628, 629, 630, 631, 632, 633	GLKS 32196*, CGN 17996, CGN 17854, CGN 17997, CGN 20578, CGN 17856, CGN 17858	6 (7)
	S. hawkesianum	haw	166, 167, 634, 635, 636, 637, 638,	GLKS 32762, GLKS 32765, CGN 17888, CGN 17889, CGN 17890, CGN 17891, CGN 17892	7 (7)

Longipedicellata	*S. hjertingii* Hawkes	hjt	349, 350, 639, 640, 641, 643	CGN 17717, CGN 17718, CGN 22369, CGN 22370, CGN 18345, cgn962453	6 (6)
Tuberosa III	*S. hondelmannii* Hawkes and Hjert.	hdm	168, 351, 644, 645, 646, 647, 648	GLKS 32852, CGN 18106, cgn961918, cgn962199, CGN 18192, CGN 18193, cgn962204	7 (7)
Tuberosa III	*S. hoopesii* Hawkes and Okada	hps	169, 650, 651, 652, 653	GLKS 32885, CGN 18363, CGN 18367, CGN 18368, CGN 18372	5 (5)
Demissa	*S. hougasii* Correll	hou	271, 272, 654, 655,	CPC 7050, CPC 2718, CGN 18339, CGN 21361	4 (4)
Yungasensa	*S. huancabambense* Ochoa	hcb	18, 170, 353, 354	PI 365359, GLKS 32441, CGN 18306, CGN 17719	4 (4)
Tuberosa II	*S. huarochiriense* Ochoa	hro	309	CIP 761224	1 (1)
Tuberosa II	*S. humectophilum* Ochoa	hmp	171	GLKS 32829	1 (1)
Piurana	*S. hypacrarthrum* Bitter	hcr	311	CIP 761259	1 (1)
Tuberosa II	*S. immite* Dunal	imt	63, 64, 172,	PI 498245, PI 365331, GLKS32819	3 (3)
Tuberosa III	*S. incamayoense* K.A. Okada and A.M. Clausen	inm	657, 658, 659, 660, 661, 662, 663, 1016	CGN 18077, CGN 21320, CGN 17874, CGN 17875, CGN 17968, cgn961363, CGN 22335, CGN 17972	8 (8)
Megistacroloba	*S. infundibuliforme* Phil.	ifd	664, 665, 666, 667, 668, 1007, 1023	CGN 17720, CGN 23063, CGN 22334, CGN 23048, cgn960696, CGN 17959, CGN 18079	7 (7)
Demissa	*S. iopetalum* (Bitter) Hawkes	iop	273, 670, 671	CPC 2922, CGN 20561, CGN 20572	3 (3)
Conicibaccata/ Tuberosa II	*S. irosinum* Ochoa	irs	66, 312, 313	PI 583305, CIP 761252, CIP 762259	3 (3)
Pinnatisecta	*S. jamesii* Torr.	jam	268*, 274, 355, 672, 673, 674	CPC 7510*, CPC 7167, CGN 18349, cgn962542, cgn960923, CGN 18346	5 (6)
Tuberosa III	*S. juzepczukii* Bukasov	juz	173, 174, 175, 176, 177	GLKS 25465, GLKS 25467, GLKS 25468, GLKS 25469, GLKS 25470	5 (5)
Tuberosa III	*S. kurtzianum* Bitter and Wittm.	ktz	275, 276, 675, 676, 677, 678, 995,	CPC 5864, CPC 5889, CGN 22338, cgn961563, CGN 23042, cgn961013, CGN 22353	7 (7)
Conicibaccate	*S. laxissimum* Bitter	lxs	178, 679	GLKS 32439, CGN 22721	2 (2)
Tuberosa II	*S. leptophyes* Bitter	lph	356, 357, 680, 682, 683, 684,	CGN 18174, CGN 18140, CGN 18173, CGN 18167, CGN 20611, CGN 18126	6 (6)

	S. leptophyes x S. sparsipilum	lph x spl	405#	CGN 20619#	1 (0)
Tuberosa I	*S. leptosepalum* Correll	lps	19	PI 607843	1 (1)
Polyadenia	*S. lesteri* Hawkes and Hjert.	les	20, 21, 358,	PI 558434, PI 558435, CGN 18337	3 (3)
Lignicaulia	*S. lignicaule* Vargas	lgl	179, 685	GLKS 32215, CGN 17723	2 (2)
Conicibaccata	*S. limbaniense* Ochoa	lmb	686	CGN 22720	1 (1)
Conicibaccata	*S. longiconicum* Bitter	lgc	68, 69, 70, 180, 687, 992	PI 208780, PI 604093, PI 604095, GLKS 35363, cgn962815, CGN 23561	6 (6)
Juglandifolia	*S. lycopersicoides* Dunal	lyc	71&, 72&, 314&	PI 558090&, PI 558091&, CIP 761078&	3 (0)
Tuberosa I	*S. macropilosum* Correll	mcp	23, 74	PI 607844, PI 607845	2 (2)
Maglia	*S. maglia* Schtdl.	mag	75, 76, 359, 688,	PI 245087, PI 558316, CGN 18064, CGN 22719	4 (4)
Maglia	*S. maglia x microdontum*	mag x mcd	406#	CGN 18250#	1 (0)
Tuberosa II	*S. marinasense* Vargas	mrn	77, 181, 182, 277, 690	PI 607884, GLKS 35430, GLKS 32281, CPC 7172, CGN 17594	5 (5)
Longipedicellata	*S. matehulae* Hjert. and T.R. Tarn	mat	192	GLKS 35364	1 (1)
Tuberosa II	*S. medians* Bitter	med	183, 691, 692, 693, 694, 695,	GLKS 32226, CGN 21349, CGN 18043, CGN18308, CGN 21343, CGN 18307	6 (6)
Megistacroloba	*S. megistacrolobum* Bitter	mga	696, 697, 699, 700	CGN 23064, CGN 17828, CGN 22347, CGN 20601	4 (4)
Megistacroloba	*S. megistacrolobum* subsp. *toralapanum* (Cárdenas and Hawkes)	tor	278, 701, 702, 703, 704, 705, 706	CPC 1773, CGN 17728, CGN 23006, CGN 18145, CGN 18146, CGN 18147, CGN 18125	7 (7)
Pinnatisecta	*S. x michoacanum* (Bitter) Rydb.	mch	185, 279	GLKS 32346, CPC 3847	2 (2)
Tuberosa III	*S. microdontum* Bitter	mcd	360, 707, 708, 958, 959, 994	CGN 17596, CGN 22382, CGN 18259, CGN 20646, CGN 18047, CGN 20597	6 (6)

Tuberosa III	*S. microdontum subsp. gigantophyllum* (Bitter) Hawkes and hjert.	gig	361, 362, 710, 711, 712, 713, 714, 715, 956, 957, 960, 961, 962, 963, 964, 965, 966, 967,	CGN 18046, CGN 18083, CGN 18199, CGN 20639, CGN 18200, CGN 17595, CGN 23050, CGN 21342, CGN 18295, CGN 23511, CGN 20586, CGN 18048, CGN 17597, CGN 18049, CGN 18084, CGN 18003, CGN 18067, CGN 22372	18
Tuberosa I	*S. minutifoliolum* Correll	min	24$	PI 583298$	1 (0)
Tuberosa II	*S. mochiquense* Ochoa	mcq	186$, 716, 717, 718, 719	GLKS 32319$, CGN 20587, CGN 18263, CGN 17731, CGN 21360	5 (4)
Morelliformia	*S. morelliforme* Bitter and G. Muench	mrl	78, 79, 187,	PI 619119, PI 545720, GLKS 32245	3 (3)
Conicibaccata	*S. moscopanum* Hawkes	msp	25, 81, 188$, 720, 721	PI 230462, PI 498159, GLKS 35366$, CGN 22355, CGN 18343	5 (4)
Tuberosa II	*S. multidissectum* Hawkes	mlt	363, 722, 723, 724, 725, 727, 728, 729, 730, 731, 732	CGN 17824, CGN 21344, CGN 18330, cgn960739, CGN 17686, CGN 17733, cgn960736, CGN 17825, cgn961613, cgn17840, cgn960967	11 (11)
Tuberosa II	*S. multiinterruptum* Bitter	mtp	190	GLKS 32431	1 (1)
Pinnatisecta	*S. nayaritense* (Bitter) Rydb.	nyr	26, 27,	PI 545825, PI 545820	2 (2)
Tuberosa III	*S. neocardenasii* Hawkes and Hjert.	ncd	193, 734	GLKS 32855, CGN 18217	2 (2)
Tuberosa III	*S. neorossii* Hawkes and Hjert.	nrs	281, 735, 736, 737, 987*	CPC 6047, CGN 18280, CGN 17599, CGN 18051, CGN 17763*	5 (5)
	S. nigrum L.	nig	968&	CGN 21367&	1 (0)
	S. ochranthum Dunal	ocr	194&	GLKS 30919&	1 (0)
Tuberosa III	*S. okadae* Hawkes and Hjert.	oka	283, 365*, 366, 367, 368, 739, 740, 741$, 742, 743, 744, 745, 746, 969, 970	CPC 7129, CGN 18000*, CGN 18109, CGN 18108, CGN 17998, CGN 18269, CGN 17999, CGN 18279$, cgn962076, cgn962078, CGN 18157, CGN 22709, CGN 18129, CGN 22703, CGN 20599	15 (14)

Tuberosa III	*S. oplocense* Hawkes	opl	747, 749, 750, 751, 752, 753, 754, 1001$, 1002, 1003, 1004, 1024, 1031, 1041, 1046, 1056, 1057, 1059	CGN 23049, cgn962217, CGN 21352, CGN 18088, CGN 18085, CGN 21319, CGN 17736$, CGN 17868, CGN 17869, CGN 17870, CGN 18087, CGN 20638, CGN 22324, CGN 22713, CGN 23798, cgn961876, cgn962541	18 (17)
Conicibaccata	*S. orocense* Ochoa	oro	28	PI 583307	1 (1)
Tuberosa II	*S. orophilum* Correll	orp	29, 83, 84, 196, 756	PI 498213, PI 498209, PI 498212, GLKS 35301, cgn962570	5 (5)
Conicibaccata	*S. otites* Dunal	oti	30	PI 570618	1 (1)
Conicibaccata	*S. oxycarpum* Schiede	oxc	32, 85, 86, 757	PI 498026, PI 545776, PI 545779, CGN 18292	4 (4)
Etuberosa	*S. palustre* Poepp.	pls	197, 198, 284, 285, 286, 759, 760, 761,	GLKS 35317, GLKS 35319, CPC 7034, CPC 1576, CPC 2451, CGN 18286, CGN 17983, CGN 18241	8 (8)
Tuberosa II	*S. pampasense* Hawkes	pam	288, 762, 763, 764	CPC 6024, CGN 962604, CGN 20575, cgn960051	4 (4)
Longipedicellata	*S. papita* Rydb.	pta	369, 370, 642*, 765, 766$, 767,	CGN 17830, CGN 17832, CGN 22385*, CGN18309, CGN 18319$, CGN17831,	5 (5)
Tuberosa I	*S. paramoense* Bitter (ex Pittier)	prm	87, 88	PI 587114, PI 604202	2 (2)
Conicibaccata	*S. paucijugum* Bitter	pcj	33, 89, 90, 199, 768	PI 561650, PI 583299, PI 561651, GLKS 35372, CGN 18050	5 (5)
Piurana	*S. paucissectum* Ochoa	pcs	91, 769$	pi590922, cgn962622$	2 (1)
Tuberosa III	*S. phureja* Juz. and Bukasov	phu	200, 201, 203, 289, 290, 371, 372, 373, 771, 772, 773, 774	GLKS 31467, GLKS 31468, GLKS 31455, CPC 4188, CPC 4414, CGN 17667, CGN 18301, CGN 18315, CGN 18342, cgn960564, CGN17668, CGN18316	12 (12)
Pinnatisecta	*S. pinnatisectum* Dunal	pnt	204, 231*, 374, 375, 775, 776, 777, 778, 779, 780, 781, 880*,	GLKS 31586, GLKS 32298*, CGN 17745, CGN 17743, CGN 17742, CGN17744, CGN 17740, CGN 23011, CGN 23012, CGN 17741, CGN 18331, CGN 18335*	10 (12)

Piurana	*S. piurae* Bitter	pur	34, 206	PI 365365, GLKS 32341	2 (2)
Polyadenia	*S. polyadenium* Greenm.	pld	207, 376, 377, 782, 783, 784, 785,	GLKS 35375, CGN 17749, CGN 17746, CGN 17748, CGN 17747, CGN 23013, CGN 23014	7 (7)
Longipedicellata	*S. polytrichon* Rydb.	plt	378, 379, 786, 787, 788, 789,	CGN 17750, CGN 17751, cgn962607, CGN 22362, CGN 22361, CGN 18318	6 (6)
Megistacroloba	*S. raphanifolium* Cárdenas and Hawkes	rap	208, 209, 291, 380, 790, 791, 792, 793, 794, 797, 798, 799, 800, 801, 976	GLKS 30637, GLKS 30644, CPC 7090, CGN 17598, cgn960772, CGN 20589, CGN 18300, CGN 18089, cgn961878, CGN 18320, CGN 17752, CGN 18033, CGN 17833, CGN 17835, CGN 17822	15 (15)
Megistacroloba / Tuberosa III	*S. raphanifolium x S. sparsipilum*	rap x spl	410#	cgn960058#	1 (0)
Tuberosa III	*S. xrechei* Hawkes and Hjert.	rch	35	PI 558227	1 (1)
Tuberosa III	*S. xrechei x S. microdontum*	rch x mcd	411#	CGN 20658#	1 (0)
Tuberosa III	*S. xruiz-lealii* Brücher	rzl	802	CGN 18117	1 (1)
Pinnatisecta	*S. xsambucinum* Rydb.	smb	92	PI 595478	1 (1)
Megistacroloba	*S. sanctae-rosae* Hawkes	sct	803, 804, 805, 806, 807, 1061	CGN 20576, CGN 22344, CGN 17910, CGN 20564, CGN 17837, cgn961619	6 (6)
Tuberosa II	*S. sandemanii* Hawkes	snd	93, 94, 808	PI 607894, PI 607895, CGN 17600	3 (3)
Conicibaccata	*S. santolallae* Vargas	san	36, 809	PI 607887, CGN 18293	2 (2)
Tuberosa II	*S. scabrifolium* Ochoa	scb	37	PI 365363	1 (1)
Demissa	*S. schenckii* Bitter	snk	212, 213, 293, 294, 810,	GLKS 30658, GLKS 30659, CPC 7165, CPC 7164, CGN 18361	5 (5)
Demissa	*S. xsemidemissum* Juz.	sem	295	CPC 7331	1 (1)
Tuberosa III	*S. xsetulosistylum* Bitter	stl	214, 811	GLKS 31014, CGN 20655	2 (2)
Juglandifolia sect .Juglandifolium	*S. sitiens* I. M. Johnst	sit	38&, 95&, 812&	pi558114&, pi558115&, cgn962632&	3 (0)
Circaeifolia	*S. soestii* Hawkes and Hjert.	sst	813	cgn962729	1 (1)
Megistacroloba	*S. sogarandinum* Ochoa	sgr	215, 315, 316, 814	GLKS 35382, CIP 761465, CIP 761586, CGN 17601	4 (4)
Piurana	*S. solisii* Hawkes	sol	216	GLKS 35383	1 (1)
Tuberosa II	*S. soukupii* Hawkes	sou	815	CGN 18061	1 (1)

Tuberosa II	*S. sparsipilum* (Bitter) Juz. and Bukasov	spl	382, 383, 384, 816, 817, 818, 819, 820, 821, 972, 973, 975, 978,	CGN 18225, CGN 18230, CGN 18154, CGN 18096, CGN 17838, CGN 18221, CGN 20653, CGN 17758, CGN 20602, CGN 18099, CGN 22702, CGN 18094, CGN 18131	13 (13)
	S. sparsipilum x S. leptophyes	spl x lph	413#	CGN 18142#	1 (0)
	S. sparsipilum x S. sucrense	spl x scr	414#	cgn960960#	1 (0)
Tuberosa III	*S. spegazzinii* Bitter	spg	217, 385, 386, 822, 823, 824, 826, 827, 828$	GLKS 32755, CGN 17759, CGN 17839, cgn960795, CGN 21318, CGN 22707, CGN 21321, CGN 23015, CGN 18034$	9 (8)
Tuberosa III	*S. stenotomum* Juz. and Bukasov	stn	218, 219, 296, 387, 388, 829,	GLKS 30762, GLKS 30732, CPC 4741, CGN 18351, CGN 20616, CGN 18161	6 (6)
Tuberosa III	*S. stenotomum subsp. goniocalyx* (Juz. and Bukasov) Hawkes	gon	220, 830$, 831, 832, 833$, 834	GLKS 32703, CGN 17621$, CGN 18314, CGN 18237, CGN 17625$, CGN 17623	6 (4)
Longipedicellata	*S. stoloniferum* Schtdl. and Bouchet	sto	221, 297, 298, 389, 390, 554*, 835, 836, 837, 838, 839, 840, 841, 842	GLKS 30512, CPC 0012, CPC 0028, CGN 17605, CGN 18332, CGN 22718*, PI 205522, CGN 17607, CGN 17606, CGN 18348, cgn962615, CGN 18334, CGN 23072, CGN 18333	13 (14)
Tuberosa III	*S. xsubandigena* Hawkes	sub	222	GLKS 30722	1 (1)
Conicibaccata	*S. subpanduratum* Ochoa	sup	223	GLKS 22873	1 (1)
Tuberosa III	*S. xsucrense* Hawkes	scr	391, 843, 844, 845, 846, 847, 848, 849, 850	CGN 18205, CGN 20628, CGN 20630, CGN 20631, CGN 18187, CGN 20634, CGN 22350, CGN 18206, CGN 18105	9 (9)
	S. sucrense x S. oplocense	scr x opl	412#	cgn962507#	1 (0)
Conicibaccata	*S. sucubunense* Ochoa	suc	39	PI 583320	1 (1)

Tuberosa III	*S. tarijense* Hawkes	tar	224, 225,280*,392, 852, 853, 854, 855, 856, 857, 858, 859, 860, 862, 863, 864, 865, 866, 867, 868, 869, 870, 871, 872, 873, 874, 875, 876, 877, 878, 879,	GLKS 31570, GLKS 31572, CPC 7208*, CGN 17861, CGN 22729, cgn962224, CGN 22714, CGN 18198, cgn960807, cgn960805, cgn960806, CGN 17975, cgn961432, CGN 21337, CGN 23795, cgn961736, CGN 17976, CGN 17974, CGN 17977, CGN 18107, cgn961128, CGN 17978, CGN 17979, cgn961441, CGN 17980, CGN 21338, CGN 17981, cgn961449 , CGN 17982, cgn961451, cgn962690	31 (31)
	S. tarijense x S. arnezii	tar x arz	418#	cgn962468#	1 (0)
	S. tarijense x S. microdontum	tar x mcd	419#	cgn960084#	1 (0)
Pinnatisecta	*S. tarnii* Hawkes and Hjert.	trn	40, 41, 226, 227, 228, 229	PI 570642, PI 498048, GLKS 32870, GLKS 35384, GLKS 35385, GLKS 32871	6 (6)
Pinnatisecta	*S. trifidum* Correll	trf	881, 882	CGN 22722, CGN 22371	2 (2)
Tuberosa III	*S. tuberosum* L.	tbr	232, 233, 234, 883, 884	GLKS 22827, GLKS 31211, GLKS 31114, CGN 22877, cgn962368	5 (5)
Tuberosa III	*S. tuberosum subsp. andigena* (Juz. and Bukasov) Hawkes	adg	235, 237, 238, 239, 240, 241, 242, 243, 885, 886, 887, 888, 889	GLKS 35027, GLKS 34963, GLKS 34867, GLKS 34149, GLKS 34509, GLKS 34523, GLKS 34630, GLKS34737, cgn960305, CGN 20610, CGN 20614, CGN 23055, cgn960441	13 (13)
Tuberosa II	*S. tuberosum* subsp. *andigena x S. curtilobum*	adg x cur	395#, 396#, 397#, 398#, 400#	CGN 18144#, CGN 18149#, CGN 18134#, cgn962564#, CGN 21355#	5 (0)
Conicibaccata	*S. tundalomense* Ochoa	tnd	244, 245, 247	GLKS 35388, GLKS 35389, GLKS 35390	3 (3)
Piurana	*S. tuquerrense* Hawkes	tuq	96, 890,	PI 590926, CGN 18353	2 (2)
Tuberosa III	*S. ugentii* Hawkes and K. A. Okada	ugt	44, 248, 249, 892,	PI 546029, GLKS 32887, GLKS 32889, CGN 18369	4 (4)

Tuberosa II	*S. velardei* Ochoa	vlr	97, 893	PI 619114, CGN 18324	2 (2)
Tuberosa III	*S. venturii* Hawkes and Hjert.	vnt	250, 894, 896, 993,	GLKS 32794, CGN 17761, cgn961508, CGN 17755	4 (4)
Tuberosa III	*S. vernei* Bitter and Wittm.	vrn	895*, 897, 898, 899, 900, 901, 902, 903, 904, 905$, 979, 980, 981, 982, 983, 984, 985, 986	CGN 17762*, CGN 22728, CGN 18111, CGN 21350, CGN 22345, CGN 18112, CGN 18114, CGN 23039, CGN 18278, CGN 17836$, CGN 18110, CGN 21315, CGN 17995, CGN 18113, CGN 18115, CGN 23516, CGN 18277, cgn963094	17 (17)
Tuberosa III	*S. vernei* subsp. *ballsii* (Hawkes) Hawkes and Hjert.	bal	906, 907, 908	CGN 17992, CGN 17993, CGN 17994	3 (3)
Tuberosa I	*S. verrucosum* Schtdl.	ver	393, 825*, 909, 910, 911, 912, 914, 915, 916, 917, 918, 919, 920, 921, 922, 923, 988, 989, 990	CGN 17768, CGN 18100*, CGN 22326, CGN 22374, CGN 17764, CGN 20567, CGN 17769, CGN 17765, CGN 17773, CGN 17771, CGN 17766, CGN 17770, CGN 17772, cgn960832, cgn960833, CGN 20566, CGN 23017, CGN 17767, CGN 17774	19 (19)
Conicibaccata	*S. violaceimarmoratum* Bitter	vio	924, 925, 926,	CGN 18296, CGN 20647, CGN 22878	3 (3)
Conicibaccata / Yungasensa	*S. violaceimarmoratum x S. yungasense*	vio x yun	420#, 421#	cgn961955#, CGN 18124#	2 (0)
Tuberosa III	*S. virgultorum* (Bitter) Cárdenas and Hawkes	vrg	927, 928, 929, 930, 931, 932$	cgn962448, CGN 17775, cgn962072, CGN 20615, cgn962077, CGN 20652$	6 (5)
Tuberosa II	*S. weberbaueri*	wbr	254, 300	GLKS 32725, CPC 6032	2 (2)
	S. weberbaueri x S. yungasense	wbr x yun	422#	CGN 20656#	1 (0)
Yungasensa	*S. yungasense* Hawkes	yun	98, 934$, 935, 936	PI 614703, CGN 18336$, CGN 20677, CGN 20676	4 (3)

	unknown species	spec	6*, 43*, 123*, 165*, 184*, 205* , 210*, 211*, 253*, 255*, 262*, 287*, 292*, 310*, 329*, 352*, 381*, 394*, 416, 533*, 601*, 649*, 726*, 796*, 891*, 933*, 974*, 998*	PI 498214*, PI 546033*, GLKS 32175*, GLKS 31512*, GLKS 35422*, GLKS 22340*, GLKS 32808*, GLKS 32809*, GLKS 32722*, GLKS 32172*, CPC 7211*, CPC 7328*, CPC 7105*, CIP 761265*, CGN 18249*, CGN 18182*, CGN 17753*, CGN 18121, CGN 20580, CGN 18137, cgn962595, CGN 18053, CGN 17685, CGN 17754, CGN 18364, CGN 18262, CGN 18052, cgn962734	28 (28)

taxonomic remarks
synonym of *S. bukasovii* (Ochoa, 1999)
classified as cultivar-group: Ajanhuiri Group within *S. tuberosum* L. (Huaman and Spooner, 2002)
synonym of *S. canasense* (Hawkes, 1990)
synonym of *S. rhomboideilanceolatum* Ochoa (Hawkes, 1990)

nomen dubium (Hawkes, 1990)
synonym of *S. stenophyllidium* (Spooner et al., 2004)
synonym of *S. iopetalum* (Spooner et al., 2004)

synonym of *S. bulbocastanum* (Spooner et al. 2004)
synonym of *S. bulbocastanum* (Spooner et al., 2004)
not from section Petota
not from section Petota

classified as a cultivar group: Chaucha Group within *S. tuberosum* L. (Huaman and Spooner, 2002)
classified as a cultivar-group: Curtilobum Group of *S. tuberosum* L. (Huaman and Spooner, 2002)

seen as a separate species *S. ehrenbergii* (Spooner et al., 2004)
not from section *Petota*
synonym of *S. stoloniferum* (Spooner et al., 2004)
synonym of *S. stoloniferum* (Spooner et al., 2004)
not from section *Petota*
not from section *Petota*

synonym of *S. leptophyes* (Ochoa, 1990)
synonym of *S. leptophyes* *(* Ochoa, 1990)
synonym of *S. leptophyes* (Ochoa, 1990)
synonym of *S. iopetalum* *(* Spooner et al., 2004)
provisional name
provisional name

classified as a cultivar-group: Juzepczukii Group within *S. tuberosum* L. (Huaman and Spooner, 2002)

S. leptosepalum is a synonym of *S. stoloniferum* (Spooner et al., 2004)
not from section Petota
synonym of *S. verrucosum* (Spooner et al. 2004)
S. matehulae is a synonym of S. hjert.ii (Spooner et al., 2004)

synonym of *S. microdontum* Bitter (van den Berg and Spooner, 1992)
synonym of *S. bukasovii* Juz. f. multidissectum (Hawkes) Ochoa
synonym of *S. stenophyllidium* (Spooner et al. 2004)
not from section Petota
not from section Petota

synonym of *S. stoloniferum* (Spooner et al. 2004)
synonym of *S. tuberosum* subsp. *andigena* (Ocoa, 1992)
classified as cultivar-group: Phureja Group within *S. tuberosum* L. (Huaman and Spooner, 2002)

synonym *of S. stoloniferum* (Spooner et al. 2004)
synonym of S. *demissum* Lindley (Spooner et al., 2004)
not from section Petota
synonym of *S. canasense (* Hawkes 1990)

classified as a cultivar-group: Stenotomum Group within *S. tuberosum* L. (Huaman and Spooner, 2002)
classified as cultivar-group: Stenotomum Group within *S. tuberosum* L. (Huaman and Spooner, 2002)
synonym of *S .tuberosum* subsp. *andigena* (Ochoa, 1992)

classified as a cultivar-group: Andigenum Group within *S. tuberosum* L. (Huaman and Spooner, 2002)

